# Comparative interactomics for virus–human protein–protein interactions: DNA viruses versus RNA viruses

**DOI:** 10.1002/2211-5463.12167

**Published:** 2017-01-04

**Authors:** Saliha Durmuş, Kutlu Ö. Ülgen

**Affiliations:** ^1^ Computational Systems Biology GroupDepartment of BioengineeringGebze Technical UniversityKocaeliTurkey; ^2^ Department of Chemical EngineeringBoğaziçi UniversityİstanbulTurkey

**Keywords:** comparative interactomics, DNA virus, next‐generation antiviral therapeutics, pathogen–host interaction, RNA virus, viral infection strategy

## Abstract

Viruses are obligatory intracellular pathogens and completely depend on their hosts for survival and reproduction. The strategies adopted by viruses to exploit host cell processes and to evade host immune systems during infections may differ largely with the type of the viral genetic material. An improved understanding of these viral infection mechanisms is only possible through a better understanding of the pathogen–host interactions (PHIs) that enable viruses to enter into the host cells and manipulate the cellular mechanisms to their own advantage. Experimentally‐verified protein–protein interaction (PPI) data of pathogen–host systems only became available at large scale within the last decade. In this study, we comparatively analyzed the current PHI networks belonging to DNA and RNA viruses and their human host, to get insights into the infection strategies used by these viral groups. We investigated the functional properties of human proteins in the PHI networks, to observe and compare the attack strategies of DNA and RNA viruses. We observed that DNA viruses are able to attack both human cellular and metabolic processes simultaneously during infections. On the other hand, RNA viruses preferentially interact with human proteins functioning in specific cellular processes as well as in intracellular transport and localization within the cell. Observing virus‐targeted human proteins, we propose heterogeneous nuclear ribonucleoproteins and transporter proteins as potential antiviral therapeutic targets. The observed common and specific infection mechanisms in terms of viral strategies to attack human proteins may provide crucial information for further design of broad and specific next‐generation antiviral therapeutics.

**Abbreviations**
DNA: deoxyribonucleic acid
dsDNA: double‐stranded DNA
dsRNA: double‐stranded RNA
EBV: Epstein–Barr virus
GO: gene ontology
HNRP: heterogeneous nuclear ribonucleoprotein
KEGG: Kyoto Encyclopedia of Genes and Genomes
kobas: KEGG Orthology Based Annotation System
PHI: pathogen–host interaction
phisto: pathogen–host interaction search tool
PPI: protein–protein interaction
RNA: ribonucleic acid
ssDNA: single‐stranded DNA
ssRNA(−): negative‐sense single‐stranded RNA
ssRNA(+): positive‐sense single‐stranded RNA
ssRNA: single‐stranded RNA

Viral infections pose ever‐increasing danger to the human beings because of emerging and reemerging diseases. Their high mutation rates enable viruses to easily develop drug resistance towards the conventional therapeutics, which mainly inhibit essential viral proteins. This makes the problem more serious, since most of the current antiviral drugs are hardly effective to the resistant virus strains. Therefore, the efforts on the next‐generation antiviral drug discovery have been focused on finding host‐oriented drug targets, which act on cellular functions essential in the virus life‐cycle 1, 2. The cellular processes are manipulated and exploited by pathogenic microorganisms through mainly physical interactions between pathogen and host proteins 3. Therefore, viral PHI networks should be investigated thoroughly in terms of the functional properties of virus‐targeted host proteins, in order to identify the cellular proteins and associated functions that are indispensable for viruses to replicate and persist within the host cell during infections. The understanding of the differences and similarities in the infection mechanisms used by different viral groups is crucial for designing broad and virus‐specific antiviral therapeutics 1, 4, 5.

Viral families are grouped based on their type of nucleic acid as genetic material, DNA or RNA 6. DNA viruses contain usually double‐stranded DNA (dsDNA) and rarely single‐stranded DNA (ssDNA). These viruses replicate using DNA‐dependent DNA polymerase. RNA viruses have typically ssRNA, but may also contain dsRNA. ssRNA viruses can be further grouped as positive‐sense (ssRNA(+)) or negative‐sense (ssRNA(−)). The genetic material of ssRNA(+) viruses is like mRNA and can be directly translated by the host cell. ssRNA(−) viruses carry RNA that is complementary to mRNA and must be converted to positive‐sense RNA using RNA polymerase before translation. An exception of this group is the Retroviruses, which replicate through DNA intermediates using reverse transcriptase despite having RNA genomes.

Owing to their very small genome sizes, viruses have restricted life capabilities and must enter into the host cells to replicate, assemble and propagate. They have evolved to develop strategies to manipulate host cell mechanisms to control their own life cycles and also to disable antiviral responses of host immune systems 4, 7, 8. Compared to DNA virus genomes, which can encode up to hundreds of viral proteins, RNA viruses have smaller genomes that usually encode only a few proteins. Owing to the size and functionality of the resulting proteome, the size and type of viral genetic materials may have great effects on the life styles of viruses within the host cells. DNA viruses have integrated large DNA sequences from the hosts to their genome, throughout the evolution. Consequently, their genomes can encode proteins with eukaryote‐originated complex functional domains and enable DNA viruses to finely exploit the metabolism of infected cells in order to promote their own replication within the cell. On the other hand, RNA virus proteins cannot exhibit such homologies with their eukaryotic counterparts, but still can communicate with host cells through complex networks of PHIs. RNA viruses have probably evolved a different strategy, i.e. they interact with the host proteins using protein‐binding motifs specific to RNA viruses 9. Consequently, it can be stated that, DNA and RNA viruses have developed some distinct infection strategies to cause generally chronic and acute infections, respectively.

Despite the availability of detailed models of virus structures, replication machineries, and patho‐physiologies, a more functional analysis of virus–host molecular interactions is required to capture a systems view of viral infection mechanisms in host cells. Considering the preliminary efforts on the high‐throughput experimental studies 4, 10, 11, 12, 13, one can state that the field of virus–human interspecies protein interactions has been developing now. Nevertheless, the aforementioned systems view of viral infection mechanisms through PHIs is still lacking 2, 14. The general focus of computational analysis of PHI data is on the common and specific behaviors of bacterial and viral pathogens during infections, by comparing their protein interactions with human 3, 15. Vidalain and Tangy (2010) reviewed RNA viruses–human protein interaction networks analyzing special infection characteristics of RNA viruses through 830 PHI data. Pichlmair *et al*. (2012) experimentally found 1681 PHI data between 70 viral proteins and 579 human proteins and then comparatively analyzed this dataset in terms of common and specific infection strategies used by DNA and RNA viruses. Here, we comparatively analyzed the current experimental PHI data belonging to DNA and RNA viruses, covering 19 033 PHIs between 1061 viral proteins and 4943 human proteins. The PHI data were obtained from phisto which is a comprehensive database of pathogen–human PPIs 16. This study presents the first comprehensive comparison between DNA and RNA viruses in terms of their infection strategies, providing an initial systems‐level understanding of viral pathogenesis through PHIs. However the results drawn from this analysis should be interpreted with caution since PHI data for lots of virus families are still scarce.

## Materials and methods

### phisto: a web‐based tool for retrieval and analysis of PHI networks

Pathogen–host interaction search tool (phisto) was developed by our group due to the lack of a comprehensive PHI database in the Web 16. It stores the up‐to‐date PHI data for all pathogen types for which experimentally‐found PPIs with human are available. phisto also provides integrated bioinformatic tools for visualization of PHI networks and topological/functional analysis of pathogen‐targeted human proteins through its user‐friendly interface (www.phisto.org). Ongoing studies on phisto are for covering experimental PHI data belonging also to other mammalian species as host organism.

### Virus–human PHI data

**Figure 1:**
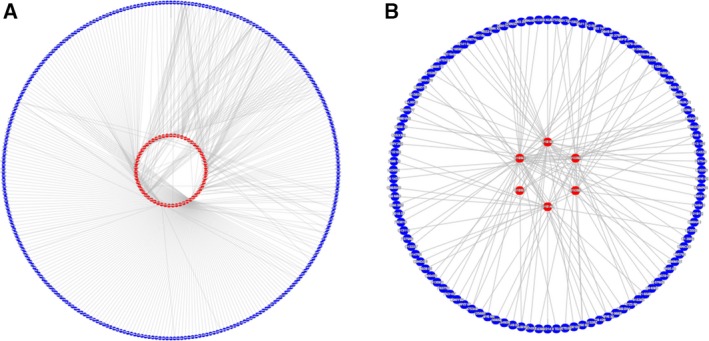
Virus–human PHI networks obtained from phisto. Red nodes are viral proteins and blue nodes are human proteins. (A) Human herpesvirus 4 (Epstein–Barr virus) – human protein–protein interaction network. (B) Influenza A virus strain WSN/1933/TS61 (H1N1) – human protein–protein interaction network.

The family‐based virus–human PHIs were downloaded from phisto using the taxonomic filtering functionality of its browse option. Twenty‐eight viral families are covered by the downloaded interspecies interactome data. Twelve families carry DNA and 16 families carry RNA as their genetic material. Retroviruses were excluded from the RNA families since they replicate through reverse transcription. Similarly, Hepadnaviruses were excluded from the DNA viruses. Consequently, PHI data belonging to 11 DNA virus families (Table 1) and 15 RNA virus families (Table 2) were obtained and used throughout the study. Two representative PHI networks, one for a DNA virus and one for an RNA virus, are in Fig. 1. All details of the PHI data for these 26 viral families are given in Data S1 and S2.

**Table 1:** Contents of DNA viruses–human PHI data

DNA virus family (genetic material)	# of species	# of strains	# of PHIs	# of pathogen proteins	# of human proteins
Adenoviridae (dsDNA)	9	13	305	43	235
Asfarviridae (dsDNA)	1	2	5	5	4
Baculoviridae (dsDNA)	1	1	1	1	1
Circoviridae (ssDNA)	1	2	4	2	3
Herpesviridae (dsDNA)	17	35	4836	391	2288
Myoviridae (dsDNA)	2	3	4	3	3
Papillomaviridae (dsDNA)	10	20	4033	91	1731
Parvoviridae (ssDNA)	2	2	60	5	56
Polyomaviridae (dsDNA)	5	6	356	18	261
Poxviridae (dsDNA)	10	19	444	78	343
Siphoviridae (dsDNA)	3	3	3	3	3
Total	61	106	10 051	640	3658

**Table 2:** Contents of RNA viruses–human PHI data

RNA virus family (genetic material)	# of species	# of strains	# of PHIs	# of pathogen proteins	# of human proteins
Arenaviridae ((−) ssRNA)	6	6	17	6	14
Arteriviridae ((+) ssRNA)	2	2	68	3	68
Birnaviridae (dsRNA)	1	1	1	1	1
Bornaviridae ((−) ssRNA)	1	1	1	1	1
Bunyaviridae ((−) ssRNA)	9	10	182	11	140
Coronaviridae ((+) ssRNA)	4	4	34	16	29
Filoviridae ((−) ssRNA)	3	4	172	6	151
Flaviviridae ((+) ssRNA)	8	30	1704	191	876
Hepeviridae ((+) ssRNA)	1	2	3	2	2
Orthomyxoviridae ((−) ssRNA)	3	49	5681	105	1623
Paramyxoviridae ((−) ssRNA)	14	22	904	42	650
Picornaviridae ((+) ssRNA)	4	7	10	9	6
Reoviridae (dsRNA)	4	8	63	11	57
Rhabdoviridae ((−) ssRNA)	5	7	27	11	22
Togaviridae ((+) ssRNA)	4	5	115	6	104
Total	69	158	8982	421	2639

### Human protein sets

A total of eight sets of human proteins interacting with viral pathogens were constructed from the PHI data to analyze the functional properties of virus‐targeted human proteins. Firstly, the sets targeted by DNA viruses (DNA viruses‐targeted set), RNA viruses (RNA viruses‐targeted set), only DNA viruses, i.e. not targeted by any RNA viruses (only DNA viruses‐targeted set), and only RNA viruses, i.e. not targeted by any DNA viruses (only RNA viruses‐targeted set) were constructed to observe the characteristics specific to DNA and RNA viruses with respect to their interactions with human proteins. For a deeper comparison between DNA and RNA viral infections, human proteins interacting with at least four DNA virus families (4‐DNA viruses‐targeted set), as well as human proteins interacting with at least four RNA virus families (4‐RNA viruses‐targeted set) were used. On the other hand, to obtain the common infection strategies of viral pathogens, sets of human proteins targeted by all viruses, i.e. targeted by DNA and/or RNA viruses (viruses‐targeted set) and the ones targeted by both DNA and RNA viruses together (DNA‐RNA viruses‐targeted set) were also analyzed. The number of virus‐targeted human proteins covered by each set is tabulated in Table 3.

**Table 3:** Virus‐targeted human protein sets

Human protein set	Targeting viruses	# of human proteins
DNA viruses‐targeted	DNA viruses	3658
RNA viruses‐targeted	RNA viruses	2639
Only DNA viruses‐targeted	Only DNA viruses	2304
Only RNA viruses‐targeted	Only RNA viruses	1285
4‐DNA viruses‐targeted	4 or more DNA virus families	60
4‐RNA viruses‐targeted	4 or more RNA virus families	84
DNA‐RNA viruses‐targeted	Both DNA and RNA viruses	1354
Viruses‐targeted	DNA and/or RNA viruses	4943

### Gene ontology enrichment analysis

Gene ontology (GO) 17 enrichment analysis of the human protein sets was performed using the bingo plug‐in of Cytoscape 18. The significance level was set to 0.05 meaning that only terms enriched with a *P*‐value of at most 0.05 were considered after an enrichment calculation with hypergeometric test and then Benjamini and Hochberg false discovery rate correction. All three GO terms (biological process, molecular function, and cellular component) were scanned to identify the terms having significant association with each virus‐targeted human protein set studied.

### Pathway enrichment analysis

Pathway enrichment analysis of the human protein sets was performed using the Web‐based tool, kobas (ver. 2.0) 19 based on information in KEGG pathway database 20. In the enrichment process, kobas platform uses hypergeometric test and Benjamini and Hochberg correction method. In this study, *P*‐value was set to 0.05 to obtain enriched human pathways for the virus‐targeted protein sets.

## Results

### Virus‐targeted human proteins

The visualization of virus–human networks (Fig. 1) provides some insights on the nature of interactions between the viral and human proteins. In a PHI network, few proteins may serve as hub nodes. These are human proteins targeted by lots of pathogen proteins; and pathogen proteins targeting lots of human proteins. This scale‐free behavior is observed in the DNA virus–human PHI network to some extent (Fig. 1A). On the other hand, in RNA viruses, very few viral proteins have roles in PHI networks because of their very small genomes. All of these RNA virus proteins usually have lots of interactions with human proteins (Fig. 1B). Therefore, in most cases, the scale‐free behavior could not be observed in RNA virus–human PHI networks. In fact, the degree distribution of the virus–human protein interaction networks could not be fitted to any model yet, mostly because of their incompleteness 21, despite some preliminary attempts to model the graph properties of PHI networks 10.

The distribution of 4943 viruses‐targeted human proteins based on their attacking virus types can be observed from the number of proteins in the human protein sets (Table 3). A considerable amount of human proteins (1354) are targeted by both DNA and RNA viruses, constituting the common viral targets. All of the virus‐targeted human proteins with the number of targeting DNA/RNA virus families can be found in Data S3. Human proteins that are highly targeted by viruses, i.e. targeted by at least total 8 viral families and the targeting virus families, are presented in Table 4. The list includes 21 such human proteins with corresponding targeting viral families.

**Table 4:** Highly targeted human proteins

Human protein	Targeting DNA virus families	Targeting RNA virus families
HNRPK – heterogeneous nuclear ribonucleoprotein K	Asfarviridae, Herpesviridae, Papillomaviridae, Poxviridae	Arteriviridae, Filoviridae, Flaviviridae, Orthomyxoviridae, Paramyxoviridae, Togaviridae
YBOX1 – nuclease‐sensitive element‐binding protein 1	Herpesviridae, Papillomaviridae, Polyomaviridae, Poxviridae	Arteriviridae, Flaviviridae, Orthomyxoviridae, Paramyxoviridae, Reoviridae, Togaviridae
EF1A1 – elongation factor 1‐alpha 1	Herpesviridae, Papillomaviridae, Poxviridae	Bunyaviridae, Filoviridae, Flaviviridae, Orthomyxoviridae, Paramyxoviridae, Reoviridae, Togaviridae
IMA1 – Importin subunit alpha‐1	Adenoviridae, Herpesviridae, Papillomaviridae, Parvoviridae, Polyomaviridae, Poxviridae	Coronaviridae, Orthomyxoviridae, Paramyxoviridae
ADT2 – ADP/ATP translocase 2	Herpesviridae, Papillomaviridae, Poxviridae	Filoviridae, Flaviviridae, Orthomyxoviridae, Paramyxoviridae, Reoviridae,Togaviridae
TBA1C – Tubulin alpha‐1C chain	Herpesviridae, Papillomaviridae, Poxviridae	Arenaviridae, Bunyaviridae, Filoviridae, Orthomyxoviridae, Paramyxoviridae, Reoviridae
ROA1 – heterogeneous nuclear ribonucleoprotein A1	Herpesviridae, Papillomaviridae, Poxviridae	Arteriviridae, Coronaviridae, Filoviridae, Flaviviridae, Orthomyxoviridae, Togaviridae
GRP78 – 78 kDa glucose‐regulated protein	Herpesviridae, Papillomaviridae, Poxviridae	Bunyaviridae, Flaviviridae, Orthomyxoviridae, Paramyxoviridae, Rhabdoviridae, Togaviridae
TBB5 – Tubulin beta chain	Herpesviridae, Poxviridae	Bunyaviridae, Filoviridae, Flaviviridae, Orthomyxoviridae, Paramyxoviridae, Reoviridae, Togaviridae
P53 – cellular tumor antigen p53	Adenoviridae, Herpesviridae, Papillomaviridae, Parvoviridae, Polyomaviridae	Flaviviridae, Orthomyxoviridae, Paramyxoviridae, Togaviridae
NPM – nucleophosmin	Adenoviridae, Herpesviridae, Papillomaviridae, Poxviridae	Flaviviridae, Orthomyxoviridae, Paramyxoviridae, Togaviridae
GBLP – guanine nucleotide‐binding protein subunit beta‐2‐like 1	Adenoviridae, Herpesviridae, Papillomaviridae, Poxviridae	Arteriviridae, Orthomyxoviridae, Paramyxoviridae, Togaviridae
TCPG – T‐complex protein 1 subunit gamma	Adenoviridae, Herpesviridae, Papillomaviridae	Arenaviridae, Bunyaviridae, Orthomyxoviridae, Paramyxoviridae, Reoviridae
EF1A3 – putative elongation factor 1‐alpha‐like 3	Herpesviridae, Papillomaviridae, Poxviridae	Bunyaviridae, Filoviridae, Orthomyxoviridae, Paramyxoviridae, Reoviridae
HNRPC – heterogeneous nuclear ribonucleoproteins C1/C2	Herpesviridae, Papillomaviridae, Poxviridae	Arteriviridae, Orthomyxoviridae, Paramyxoviridae, Rhabdoviridae, Togaviridae
TCPE – T‐complex protein 1 subunit epsilon	Adenoviridae, Herpesviridae	Arenaviridae, Bunyaviridae, Filoviridae, Orthomyxoviridae, Paramyxoviridae, Reoviridae
HS90B – heat shock protein HSP 90‐beta	Herpesviridae, Poxviridae	Bunyaviridae, Filoviridae, Flaviviridae, Orthomyxoviridae, Paramyxoviridae, Togaviridae
HNRH1 – heterogeneous nuclear ribonucleoprotein H	Herpesviridae, Papillomaviridae	Arteriviridae, Bunyaviridae, Filoviridae, Flaviviridae, Orthomyxoviridae, Paramyxoviridae
TBA1A – Tubulin alpha‐1A chain	Herpesviridae, Poxviridae	Arenaviridae, Bunyaviridae, Filoviridae, Orthomyxoviridae, Paramyxoviridae, Reoviridae
HNRPF – heterogeneous nuclear ribonucleoprotein F	Herpesviridae, Papillomaviridae	Arteriviridae, Bunyaviridae, Filoviridae, Flaviviridae, Orthomyxoviridae, Paramyxoviridae
ROA2 – heterogeneous nuclear ribonucleoproteins A2/B1	Herpesviridae, Poxviridae	Arteriviridae, Filoviridae, Flaviviridae, Orthomyxoviridae, Paramyxoviridae, Togaviridae

### Functional analysis of the virus‐targeted human proteins

#### GO enrichment analysis results

The enriched GO process terms can be used to point out the human processes that are attacked by DNA/RNA viruses. All enriched GO process, function and component terms for each human protein set are available in Data S4. Special attention should be given to the results of sets of human proteins interacting with only DNA virus proteins and only RNA virus proteins (Table 5) to retrieve specific attack strategies of these two different virus types. Enriched GO processes for the human proteins targeted highly by multiple viral families, 4‐DNA viruses‐targeted set and 4‐RNA viruses‐targeted set (Table 6) may reflect more specificity to infection mechanisms of the corresponding virus types. On the other hand, the results of human proteins interacting with both DNA and RNA viruses (Table 7) are also important to highlight common infection mechanisms shared by the two types of viruses.

**Table 5:** Top 20 enriched GO process terms in only DNA viruses‐targeted and only RNA viruses‐targeted human protein sets

Enriched GO process terms in only DNA viruses‐targeted set	*P*‐value	Enriched GO process terms in only RNA viruses‐targeted set	*P*‐value
Cellular process	4.94E‐26	Gene expression	2.53E‐15
Cellular macromolecule metabolic process	2.86E‐19	Cellular process	4.75E‐14
Cellular metabolic process	4.61E‐19	RNA processing	9.57E‐11
Positive regulation of cellular process	1.47E‐18	RNA splicing	2.68E‐09
Positive regulation of biological process	4.49E‐16	RNA metabolic process	2.68E‐09
Macromolecule metabolic process	1.34E‐15	mRNA metabolic process	2.81E‐09
Cellular component organization	1.99E‐14	Nucleobase, nucleoside, nucleotide and nucleic acid metabolic process	1.07E‐08
Metabolic process	1.26E‐13	Cellular metabolic process	1.25E‐08
Primary metabolic process	1.92E‐13	mRNA processing	2.63E‐08
Cellular protein metabolic process	4.14E‐12	RNA localization	4.45E‐08
Post‐translational protein modification	5.25E‐11	Nucleic acid metabolic process	7.77E‐08
Organelle organization	1.88E‐10	Nucleic acid transport	8.10E‐08
Protein modification process	3.30E‐10	RNA transport	8.10E‐08
Cell cycle	3.30E‐10	Establishment of RNA localization	8.10E‐08
Interspecies interaction between organisms	7.65E‐10	Cellular component organization	3.25E‐07
Macromolecule modification	3.28E‐09	Cellular nitrogen compound metabolic process	3.25E‐07
Regulation of molecular function	8.70E‐09	Nucleobase, nucleoside, nucleotide and nucleic acid transport	4.02E‐07
Regulation of cell cycle	9.08E‐09	Metabolic process	7.33E‐07
Nucleobase, nucleoside, nucleotide and nucleic acid metabolic process	1.05E‐08	mRNA transport	7.69E‐07
Positive regulation of apoptosis	1.06E‐08	Macromolecule localization	1.39E‐06

**Table 6:** Top 20 enriched GO process terms in 4‐DNA viruses‐targeted and 4‐RNA viruses‐targeted human protein sets

Enriched GO process terms in 4‐DNA viruses‐targeted set	*P*‐value	Enriched GO process terms in 4‐RNA viruses‐targeted set	*P*‐value
Interspecies interaction between organisms	1.53E‐08	Cellular macromolecular complex assembly	9.83E‐11
Positive regulation of biological process	2.25E‐06	Cellular macromolecular complex subunit organization	3.42E‐10
Multi‐organism process	2.50E‐05	Nucleosome assembly	1.78E‐09
Positive regulation of cellular process	3.77E‐05	Chromatin assembly	2.18E‐09
Modulation by host of viral transcription	1.59E‐04	Protein‐DNA complex assembly	2.89E‐09
Modulation of transcription in other organism involved in symbiotic interaction	1.59E‐04	Nucleosome organization	2.89E‐09
Modulation by host of symbiont transcription	1.59E‐04	DNA packaging	2.78E‐08
Anatomical structure formation involved in morphogenesis	2.82E‐04	Macromolecular complex assembly	3.33E‐08
Protein import into nucleus	4.12E‐04	Chromatin assembly or disassembly	4.52E‐08
Nuclear import	4.51E‐04	Protein folding	4.66E‐08
Nucleocytoplasmic transport	5.50E‐04	Macromolecular complex subunit organization	6.99E‐08
Nuclear transport	5.50E‐04	Cellular process	6.99E‐08
Modification by host of symbiont morphology or physiology	6.42E‐04	RNA splicing	6.99E‐08
Regulation of viral transcription	6.42E‐04	DNA conformation change	7.12E‐08
Protein localization in nucleus	7.63E‐04	Gene expression	1.43E‐07
Cellular process	8.59E‐04	Cellular component assembly	3.93E‐07
Regulation of protein modification process	1.04E‐03	Cellular component biogenesis	5.46E‐07
Blood vessel development	1.05E‐03	Cellular macromolecule metabolic process	9.59E‐07
Positive regulation of viral reproduction	1.07E‐03	Translational elongation	9.59E‐07
Vasculature development	1.07E‐03	Response to unfolded protein	1.25E‐06

**Table 7:** Top 20 enriched GO process terms in DNA‐RNA viruses‐targeted human protein set

GO process term	*P*‐value
Translational elongation	3.15E‐62
Cellular macromolecule metabolic process	1.21E‐51
Translation	2.28E‐46
Gene expression	4.62E‐46
Interspecies interaction between organisms	6.88E‐46
Cellular process	4.86E‐43
Macromolecule metabolic process	6.46E‐42
Cellular metabolic process	8.78E‐39
Cellular macromolecule biosynthetic process	4.41E‐34
Cellular protein metabolic process	4.86E‐34
Macromolecule biosynthetic process	7.66E‐33
Cellular component biogenesis	4.07E‐32
Macromolecular complex assembly	1.95E‐31
Macromolecular complex subunit organization	1.64E‐29
Primary metabolic process	2.32E‐29
Cellular macromolecular complex assembly	7.09E‐29
Cellular component assembly	7.17E‐29
Cellular macromolecular complex subunit organization	7.64E‐28
Intracellular transport	7.41E‐27
Metabolic process	3.67E‐26

#### Pathway enrichment analysis results

Enriched pathway terms for five specific human protein sets are listed in Tables 8, 9, 10, presenting the certain characteristics of DNA and RNA viruses attack strategies. Similar to GO enrichment analysis results, these human protein sets are only DNA viruses‐targeted and only RNA viruses‐targeted (Table 8), 4‐DNA viruses‐targeted and 4‐RNA viruses‐targeted (Table 9), and DNA‐RNA viruses‐targeted (Table 10). Pathway enrichment analysis results are provided in Data S5 for all of eight virus‐targeted human protein sets under investigation.

**Table 8:** Enriched pathway terms in only DNA viruses‐targeted and only RNA viruses‐targeted human protein sets

Enriched pathway terms in only DNA viruses‐targeted set	*P*‐value	Enriched pathway terms in only RNA viruses‐targeted set	*P*‐value
Cell cycle	2.55E‐04	RNA transport	1.18E‐11
Oxidative phosphorylation	1.04E‐02	Spliceosome	6.43E‐08
Huntington's disease	1.09E‐02	mRNA surveillance pathway	5.36E‐04
Viral carcinogenesis	2.23E‐02	Pathogenic *Escherichia coli* infection	1.41E‐02
p53 signaling pathway	2.59E‐02	Aminoacyl‐tRNA biosynthesis	2.16E‐02
TNF signaling pathway	3.49E‐02	Ribosome biogenesis in eukaryotes	3.05E‐02
Small cell lung cancer	3.80E‐02		
Notch signaling pathway	3.80E‐02		

**Table 9:** Enriched pathway terms in 4‐DNA viruses‐targeted and 4‐RNA viruses‐targeted human protein sets

Enriched pathway terms in 4‐DNA viruses‐targeted set	*P*‐value	Enriched pathway terms in 4‐RNA viruses‐targeted set	*P*‐value
Epstein–Barr virus infection	1.06E‐03	Systemic lupus erythematosus	1.79E‐06
Viral carcinogenesis	4.96E‐03	Alcoholism	1.74E‐05
		Pathogenic *Escherichia coli* infection	1.69E‐04
		Ribosome	1.14E‐03
		Protein processing in endoplasmic reticulum	1.94E‐03
		Antigen processing and presentation	5.20E‐03
		Spliceosome	7.74E‐03
		Legionellosis	2.04E‐02
		Phagosome	2.52E‐02

**Table 10:** Enriched pathway terms in DNA‐RNAviruses targeted human protein set

Pathway term	*P*‐value
Ribosome	1.58E‐22
Epstein–Barr virus infection	1.23E‐11
Proteasome	1.23E‐11
Protein processing in endoplasmic reticulum	4.81E‐08
Spliceosome	8.68E‐07
Viral carcinogenesis	3.04E‐06
Herpes simplex infection	5.69E‐05
RNA transport	1.26E‐04
Systemic lupus erythematosus	4.82E‐03
Pathogenic *Escherichia coli* infection	9.56E‐03
Influenza A	1.23E‐02
Toxoplasmosis	1.23E‐02
Small cell lung cancer	1.46E‐02
Hepatitis B	1.76E‐02
mRNA surveillance pathway	1.78E‐02
Hepatitis C	1.82E‐02
Measles	1.93E‐02
Alcoholism	4.13E‐02
Chronic myeloid leukemia	4.95E‐02

## Discussion

Most of the current antiviral therapeutics act for inhibiting specific viral proteins, e.g. essential viral enzymes. Unfortunately, this approach has been ineffective because of drug resistance developed by viruses, especially in the case of RNA viruses which can mutate very rapidly. The next‐generation antiviral therapeutics are emerging which target host proteins required by the pathogens, instead of targeting pathogen proteins. If these host factors are indispensable for pathogens, but not essential for host cells, their silencing may effectively inhibit infections without developing drug resistance rapidly 1, 21, 22. Another alternative approach is to inhibit the interactions between these host factors and pathogen proteins, instead of targeting the proteins 23. The development of these novel strategic therapeutic approaches against infectious diseases raises the need for enlightening the infection mechanisms through PHIs, in order to identify putative host‐oriented anti‐infective therapeutic targets. To understand the complex mechanisms of infections, computational analysis of underlying protein interaction networks may serve crucial insights to develop non‐conventional solutions 2, 14, 24. This study of computational analysis of virus–human interactomes aims to provide initial insights on the infection mechanisms of DNA and RNA viruses, comparatively, through the observation of the characteristics of human proteins interacting with viral proteins. The common and special infection strategies of DNA and RNA viruses found here may lead to the development of broad and specific next‐generation antiviral therapeutics.

### Highly targeted human proteins

As the main viral infection strategy, all viruses manipulate cellular processes to proliferate within the host. Therefore, viral proteins highly interact with human proteins functioning in cell cycle, human transcription factors to promote viral genetic material transcription, nuclear membrane proteins for transporting viral genetic material across the nuclear membrane, and also regulatory proteins for translation and apoptosis 3, 15, 25, 26. We identified human proteins that are highly interacting with viral proteins, sequentially based on the total number of targeting virus families (Table 4). The list includes the top viral targets which interact with multiple viral families, within the most comprehensive PHI data. Some of these human proteins were previously reported as targets for multiple viruses, i.e. P53, NPM, ROA2, GBLP, and HNRPK 3, 15.

Our analyses revealed that there are six heterogeneous nuclear ribonucleoproteins (HNRPs) in the highly targeted human proteins list (HNRPK, ROA1, HNRPC, HNRH1, HNRPF, ROA2). HNRPs are RNA‐binding proteins, which function in processing heterogeneous nuclear RNAs into mature mRNAs and in regulating gene expression. Specifically, they take role in the export of mRNA from the nucleus to the cytoplasm. They also recruit regulatory proteins associated with pathways related to DNA and RNA metabolism 27, 28. Being targeted by multiple viruses, HNRPU was reported as a hotspot of viral infection, and proposed as a potential antiviral human protein 4. In the present study, HNRPU is found to be targeted by five viral families (see Data S3). Our data additionally indicate several other HNRPs, targeted by viral proteins (see Data S1–S3). For all virus‐targeted HNRPs, the number of targeting RNA virus families is found to be higher than that of DNA virus families (see Data S3), revealing that they may play crucial roles in viral RNA processing. The protein family of HNRPs may serve as host‐oriented antiviral drug targets.

Moreover, our analyses also reflected that proteins functioning in transport and localization related processes within the cell are targeted highly by both DNA and RNA viruses, i.e. IMA1, ADT2, TCPG, and TCPE. IMA1 (Karyopherin alpha 2, KPNA2) functions mainly in nuclear import as an adapter protein for nuclear receptor KPNB1 (Karyopherin beta 1). Interacting with IMA1 enables viruses to enter the nucleus and consequently to use the host's transcriptional machinery. Besides, viruses may interact with IMA1 in order to inhibit the host antiviral response, since nuclear import factors regulate the transport of innate immune regulatory proteins to the nucleus of cells to activate the antiviral response 3, 29, 30, 31. The transmembrane transporter activity of ADT2 is responsible for the exchange of cytoplasmic ADP with mitochondrial ATP across the mitochondrial membrane, serving crucial roles in metabolic processes 32. Attacking to human metabolic processes was reported as a common infection strategy of bacteria and viruses 15. The proteins, TCPG and TCPE are responsible for RNA localization activity and our results reveal that they are targeted by larger number of RNA families (Table 4). Highly targeted transporter proteins should be investigated further for their potential to be next‐generation antiviral target, because of their crucial roles in viral life cycle within the host organism.

EF1A1 and EF1A3 function as translation elongation factors in protein biosynthesis. EF1A proteins promote the GTP‐dependent binding of aminoacyl‐tRNA to the A‐site of ribosomes during protein biosynthesis with a responsibility of achieving accuracy of translation 33. Translation elongation factors were reported as targets for viruses, in early studies 34, 35, 36. Since they are essential components of the cellular translational machinery, viruses interact with them for biosynthesis of viral proteins within the host cell. We found translational elongation as the top biological process, commonly targeted by both DNA and RNA viruses (Table 7).

Interacting with human transcription factors was reported as one of the main viral infection strategies 3, 15. Among the highly targeted human proteins, YBOX1 and P53 have transcription factor activity. Both of these proteins are multifunctional. YBOX1 functions in transcription of numerous genes, as a transcription factor. It also contributes to the regulation of translation. On the other hand, P53 is the famous tumor supressor acting as an activator for apoptotic cell death. Apoptosis is a very crucial process during the viral infection progress, and should be strategically controlled by viruses for a successful viral infection. Apoptosis is an innate immune response to viral infection. In the early stage of viral life cycle in the host cell, apoptosis is inhibited by corresponding virus–human protein interactions. After completion of transcription and translation of viral genetic material, viruses try to induce apoptosis to assist virus dissemination 37, 38, 39.

Among the highly targeted human proteins in Table 4, EF1A1, ADT2, TBA1C, GRP78, TBB5, P53, TCPG, HS90B, and TBA1A were found as drug targets listed in DrugBank 40. However, only ADT2, GRP78, TBB5, P53, and TBA1A are approved for commercial drugs. Nevertheless, no antiviral therapeutic usage is available for these drug targets yet. Above‐mentioned human proteins; ribonucleoproteins, proteins functioning in intracellular transport and localization, translation elongation factors and transcription factors require further investigation for their potential for serving as antiviral drug targets.

### Targeted human mechanisms

Gene ontology and pathway enrichment analyses of pathogen‐targeted host proteins are widely used in bioinformatic analysis of PHI networks to understand the attack strategies of pathogens 3, 4, 15, 41, 42 as well as in verification of computationally predicted PHIs 43. Additionally, GO and pathway terms are widely used as features in computational PHI prediction studies 44, 45.

Our observation of the enriched GO process terms for human proteins targeted by only DNA viruses (Table 5) may lead to the conclusion that DNA viruses have specifically evolved to be able to attack human cellular and metabolic processes simultaneously, during infections. Using this PHI mechanism, DNA viruses can finely exploit the cellular and metabolic mechanisms of infected cells to their own advantage, generally resulting in chronic infections in human. On the other hand, GO process terms enriched in human proteins targeted by only RNA viruses are mostly related to RNA processing, intracellular transport and localization within the cell (Table 5). It was reported that RNA viruses extensively target human proteins that are involved in RNA metabolism and also protein and RNA transport to promote viral RNA processing for a successful infection 4.

Further investigation of the enriched processes of human proteins attacked by multiple DNA viruses (Table 6) pointed out their high preference to target cellular processes. It was reported that DNA viruses tend to target crosstalking human proteins linking the cell cycle with either transcription or chromosome biology, with a possible aim of promoting viral replication instead of cellular growth 4. For the RNA viruses, we found that the human proteins attacked by multiple RNA virus families are enriched in specific processes within the cellular mechanisms (Table 6). All viruses need host's transcriptional machinery for viral genetic material transcription.

In the case of human proteins targeted by both DNA and RNA viruses, the *P*‐values of the enriched GO process terms are very low, indicating statistically strong results (Table 7). The most highly‐targeted human process is translational elongation. Translational control of viral gene expression in eukaryotic hosts was reported repeatedly 46, 47, 48. Here, we presented translational elongation as the top GO process term enriched in human proteins targeted by both DNA and RNA viruses within the current experimental PHI data. The remaining list includes cellular and metabolic processes, which can be considered as targets of both virus types. Based on these observations, we can state that the common viral infection strategy is to target human proteins functioning within the processes of gene expression and protein synthesis, simply because of the lack of their own such machineries. All viruses depend on the cellular mechanisms for these processes and they recruit host ribosomes for translation of viral proteins.

A comparative investigation of the enriched pathway terms for human protein sets targeted by only DNA viruses and by only RNA viruses (Table 8) reveals additional support for the different infection strategies of these viral groups. There is no common term in these two lists of enriched human pathways. Cell cycle pathway targeted by only DNA viruses and RNA‐related pathways targeted by only RNA viruses, provide parallel results with GO enrichment analyses. The enriched pathway terms in 4‐DNA viruses‐targeted human protein set are only Epstein–Barr virus (EBV) infection and viral carcinogenesis (Table 9). EBV is a species of DNA virus family Herpesviridae, which constitute nearly half of the DNA viruses–human PHI data (Table 1). On the other hand, it is estimated that 15% of all human tumors are caused by viruses, mainly DNA viruses, i.e. Herpesviruses and Papillomaviruses 49. The pathway enrichment analysis of 4‐RNA viruses‐targeted set brings the terms of protein processing and immune system related terms forward (Table 9). Finally, for the common targets of two virus types, we obtained ribosome term enriched with a very small *P*‐value (Table 10). Both viruses use host ribosome for viral protein synthesis.

## Conclusions

In this study, an initial system‐level understanding of viral infection mechanisms through PHI networks was pursued by comparing DNA and RNA viruses, aiming to provide a framework for further investigations of infection mechanisms in the light of more precise information on pathogen–host systems in the near future. Ongoing studies and increasing amounts of experimentally‐verified PHI data will further improve our understanding of the interplay between pathogens and human and hopefully identify novel and effective therapeutics for infectious disesases.

## Author contributions

SD and KÖÜ conceived the study. SD performed the study. SD and KÜÖ prepared the manuscript.

## Supporting information


**Data S1.** DNA viruses–human PHI data.

**Data S2.** RNA viruses–human PHI data.

**Data S3.** Number of targeting DNA/RNA virus families for each targeted human proteins.

**Data S4.** GO enrichment results for the human protein sets.

**Data S5.** Pathway enrichment results for the human protein sets.
